# The Role of Forage Quantity and Quality in the Migration and Diet of a Northern Ungulate During Their Neonatal Period

**DOI:** 10.1002/ece3.73454

**Published:** 2026-04-08

**Authors:** Sebastian Buitrago Gutierrez, Lee J. Hecker, Edward W. Bork, Mark A. Edwards, Scott E. Nielsen

**Affiliations:** ^1^ Department of Renewable Resources University of Alberta Edmonton Alberta Canada; ^2^ Maine Agricultural and Forest Experiment Station University of Maine Orono Maine USA; ^3^ Department of Agricultural, Food and Nutritional Science University of Alberta Edmonton Alberta Canada; ^4^ Office of the Chief Scientist, Alberta Environment and Protected Areas Edmonton Alberta Canada

**Keywords:** forage quality, forage quantity, migration, wood bison

## Abstract

Migration has evolved as a strategy to maximize survival and reproductive success, driven by the search for better resources and/or predator avoidance. For ungulates at high latitudes, the search for higher quality and more abundant forage has been proposed as one of the best explanations of seasonal migrations. However, responses vary among populations, species, and ecosystems. In this study, we examine the forage resources associated with an annual migration of a herd of wood bison ( *Bison bison athabascae* ) in northeast Alberta, Canada. Timing of this migration corresponds to the neonatal period in late spring during green up when females have a higher nutritional demand imposed by gestation and maternal care of neonates. The objective of this work was to assess how forage quantity (i.e., biomass) and quality (i.e., crude protein and metabolizable energy, ME) differed between the herd's core and neonatal ranges while evaluating differences in their diet. Bison diets during winter in the core range were dominated by graminoids and shrubs, while shrubs and forbs were predominant in diets within the neonatal range from late spring through summer. Overall, the neonatal range during spring had significantly greater biomass (*p* < 0.001) of shrubs and forbs compared with the core range during the same season, being 1.7 and 3.8 times higher, respectively. The neonatal range also had comparatively more crude protein and ME (*p* < 0.001) during this season, with crude protein being 2.7 and 3.2 and ME being 3.0 and 3.7 times greater than in the core range for shrubs and forbs, respectively. Conversely, the core range had higher biomass, crude protein, and ME (*p* < 0.001) available for graminoids compared with the neonatal range during spring. However, graminoids are not particularly common in their diet during the migration period. Our results suggest that bison migration to the neonatal range, coincident with higher quantity and quality of forage, is important for female wood bison, particularly given their increased post‐parturition demands for energy and protein during this critical neonatal period.

## Graphical abstract

In this article, we examined the differences in forage quantity and quality between two defined ranges of a wood bison population as an attempt to identify possible factors influencing their migration during the neonatal period. We found significant differences in the forage quantity and quality between ranges, suggesting a link between these factors with bison migration in late spring, which aligns with the higher nutritional requirements that female bison are experiencing during this time of the year.

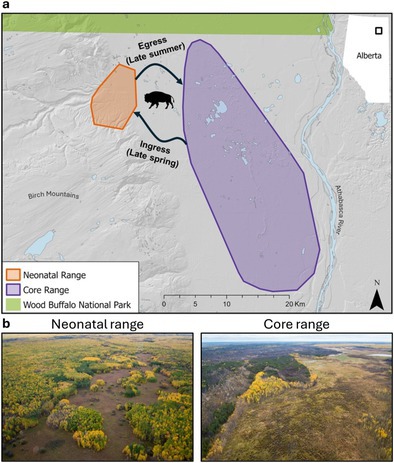

## 1 Introduction

Migration has evolved as a strategy to balance predation and the spatiotemporal variability of resources, with the underlying principle that organisms distribute themselves to maximize their survival and reproductive success (Boyce 1979; Orians and Wittenberger 1991; Fryxell and Sinclair 1988). For large‐bodied herbivores, migration is predominantly influenced by the search for more abundant and higher quality forage, particularly in temperate environments with marked seasonality (Fretwell 1972; Dupke et al. 2017; Sigrist et al. 2022). When moving to a new location inside their home range, herbivores inevitably trade‐off between forage quantity and quality (MacArthur and Pianka 1966; Van der Wal et al. 2000). While high amounts of forage can improve short‐term food intake, it can also constrain forage‐processing time due to lower digestibility of highly fibrous materials (Spalinger and Hobbs 1992; Wilmshurst et al. 1999). Therefore, to maximize energy intake, individuals often search for new vegetative growth that is rich in nutrients, such as nitrogen content (i.e., protein), but low in fiber (Fryxell 1991). One proposed mechanism regulating seasonal migration in ungulates is to provide timely access to new nutrient‐rich forages that are unique to different areas (McNaughton 1985; Bischof et al. 2012; Aikens et al. 2017), although this may vary among populations, species, and ecosystems (Mysterud et al. 2011; Geremia et al. 2019; Laforge et al. 2021).

Forage nutritional value, particularly in terms of protein and digestible energy, declines as the growing season progresses and plants mature, peaking during the spring growing season and steadily diminishing through summer and fall, reaching its lowest levels in winter (Van Soest 1982; Langvatn and Hanley 1993; Cook et al. 2016). Although graminoids, forbs, and shrubs all undergo seasonal changes in quality, they each have a distinct nutrient composition (Lee 2018) that influences their selection by ungulates. During the spring and summer, graminoids generally contain higher levels of cellulose, hemicellulose and lignin (i.e., fiber), but have lower levels of protein compared with shrubs and forbs (Robbins 1993; Moore and Jung 2001; Lee 2018). Since energy is derived from the digestion of food components (i.e., protein, carbohydrates, fat), the lower digestibility of graminoids also limits the amount of energy they can provide (Bliss 1962; Robbins 1993). As a result, ungulates aiming to maximize their energy and protein intake are more likely to choose shrubs and forbs over graminoids when given the choice, particularly during times of the year when quality differences among these growth forms are pronounced (Hofmann 1989; Hecker et al. 2021).

Ungulates living in temperate environments, where seasonality exposes them to harsh weather conditions, typically exhibit high nutritional requirements while also having short periods of favorable foraging conditions to meet their metabolic needs and attain mass (Lawler and White 2003; Lovegrove 2000; Strickland et al. 2005). This is particularly true for females, as gestation and lactation impose high protein and energetic demands (Thomas 1971; Oftedal 1985; Bowyer 1991). Female ungulates can experience up to 50% and 215% increases in their energetic requirement while gestating and lactating, respectively, with the highest requirements occurring from late winter into mid‐summer (Oftedal 1985; Pekins et al. 1998). Since graminoids, which are high in fiber but low in protein, are the predominant source of food in winter, female ungulates should select for shrubs and forbs during the spring and summer when nutritious new growth becomes available (White 1983; Gordon and Illius 1989; Lee 2018).

The Ronald Lake wood bison ( *Bison bison athabascae* Rhoads 1897) herd (RLBH) is a small ungulate population (~270 individuals) located in northeastern Alberta, Canada. The herd is of high conservation value due to its disease‐free status, distinctive genetic structure among Alberta's wood bison herds, and their cultural importance to regional Indigenous communities (Shury et al. 2015; Ball et al. 2016; Nishi 2017). The population is listed as “Threatened” under the Alberta Wildlife Act (Government of Alberta 2023) and Canada's Species at Risk Act (Government of Canada 2023), and have been the focus of research and management during the last decade due to oil sand exploration and proposed oil sands mining that overlaps part of the herd's core range (Sheppard et al. 2021; Hecker et al. 2021, 2023). The herd exhibits an annual migration in late spring/early summer (mid to late‐May) to an 82‐ha upland meadow complex west of their core range, near the northeastern base of the Birch Mountains (hereafter “neonatal range”; Hecker et al. 2024). Contrary to migrations exhibited by other American bison ( *Bison bison* ) populations between summer and winter ranges (e.g., Plumb et al. 2009; Geremia et al. 2019), the RLBH migrates a short distance (~28‐km) over a short period (~6‐days) to their neonatal range, and migrate back to their core range about 5 to 6 weeks later (Hecker et al. 2024). The reasons for this migration are unknown; however, the timing corresponds with the neonatal period and coincides with the active spring green up of new vegetative growth.

We sought to understand what factors influence the seasonal migration of the RLBH (hereafter, “the herd”) from the core range to the spring neonatal range during the neonatal period. Dewart (2023) found that predation on the herd by wolves ( *Canis lupus* ) in this area was limited during that time period. We therefore concentrated on examining bottom‐up differences in forage quantity and quality between the two seasonal ranges. Specifically, our objectives were to: (1) compare bison diet contents using fecal samples collected in the core and neonatal ranges across different times of the year; and (2) test whether forage quantity and quality differ between the two ranges. We hypothesized that: (1) the herd's diet will be mainly composed of graminoids in their core range during winter, as they are the most available forage. In the neonatal range from early spring to early summer, bison diets should shift to new vegetative growth of shrubs and forbs due to its high nutritional value, and by the end of summer in the core range, the diet should be more balanced among shrubs, forbs and graminoids; (2) given the higher nutritional demands during the neonatal period, we also hypothesized that the neonatal range will have greater quantity (i.e., biomass) and quality (i.e., protein and energy) of forage compared with the core range, thus helping to explain the migratory behavior. This work builds on previous studies of the herd's habitat selection, but with a focus on the neonatal period and the goal of informing conservation and management decisions on critical habitat for the herd during this brief, but important period.

## 2 Methods

### 2.1 Study Area

**FIGURE 1:**
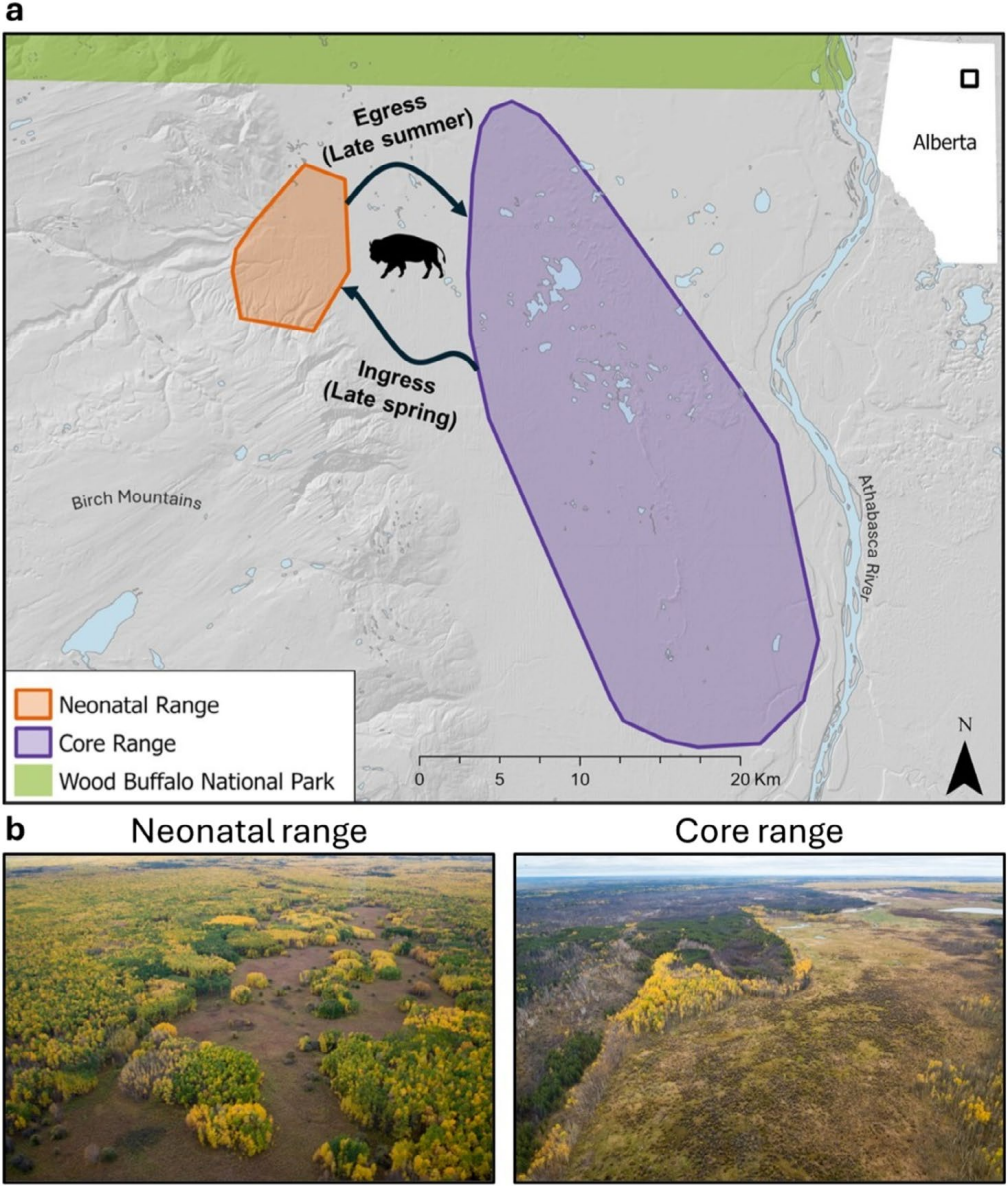
Ronald Lake wood bison herd study area in northeastern Alberta, Canada. Generalized maps of the herd's ranges and movements (a), and aerial photographs (*Source:* S. Nielsen) of the two ranges illustrating the representative conditions during early fall (b).

The herd occupies an area centered on Ronald Lake in northeastern Alberta, Canada. Our study area encompasses the herd's core home range and their neonatal range, extending from the southeastern corner of Wood Buffalo National Park in the north, south into Alberta's oil sands region, east to the Athabasca River and west to the Birch Mountains (Figure 1a; DeMars et al. 2020). Elevation ranges from 240 to 300 m above sea level with the climate characterized as northern continental, having short and warm summers, and long and cold winters (Downing and Pettapiece 2006). The study area is located within the Boreal Plains Ecoregion and presents a mosaic of ecosystems dominated by deciduous, coniferous, and mixedwood forest in the uplands, with marshes and peatlands in the lowlands (Downing and Pettapiece 2006). While these ecosystems are found in the core range, the neonatal range is characterized by a long, continuous (~2‐km length by ~0.5‐km width) upland shrubby meadow that is surrounded by upland deciduous and mixed forests, with little coverage of wetland/peatland ecosystems (Figure 1b). In the core range, forests are dominated by trembling aspen ( *Populus tremuloides* ), white spruce ( *Picea glauca* ), and jack pine ( *Pinus banksiana* ), and wetlands and marshes have abundant sedges (*Carex* spp.) and grasses from the Poaceae family. While in the neonatal range, the meadow ecosystem is mainly composed of prickly rose ( *Rosa acicularis* ), wild red raspberry ( *Rubus idaeus* ), fly honeysuckle ( *Lonicera villosa* ), and bluejoint grass ( *Calamagrostis canadensis* ).

### 2.2 Study Design

We assessed bison diets and available forage characteristics based on data collected in spring and summer of 2018, 2019, 2020, and 2023. For the core range from 2018 to 2020, we selected random female bison locations from GPS telemetry data, as well as random sites, focusing on the area from Ronald Lake in the north to the southern extent of the herd's range (Hecker et al. 2023). In 2023, we sampled the neonatal range using a stratified random design to select locations based on their relative position to the meadow complex and the relative intensity of use as determined by the history of GPS telemetry locations because the intensity of use in the area was high. We focused on the meadow complex as the number and concentration of GPS telemetry locations there was greatest. At each sample site in both ranges, we used a quadrat (0.0625‐m^2^) centered at confirmed bison locations (i.e., fecal pats, foraged vegetation, bedding signs) or at the original coordinates if no bison sign was found to collect data on bison diets, available forage biomass and macronutrient content (see Appendix S1 for photos illustrating the sampling protocol and study sites).

GPS telemetry locations came from collar data from adult female wood bison captured and marked between 2013 and 2023 by Alberta Environment and Protected Areas. The procedures used to capture and collar bison were approved by the Alberta Wildlife Animal Care Committee (permits nos. 51,244, 53,893, 54,723, and 55,748). GPS radio collars were set to record locations every 90 min, and animal locations were filtered for errors by removing locations with low accuracy (dilution of precision > 5; Bjorneraas et al. 2010) and where individuals moved beyond the range a bison can move in a 90‐min interval.

### 2.3 Seasonal Diet Content

We collected fresh bison fecal samples during 2018, 2019, and 2023 in a 15‐m radius around each survey bison location. If multiple fecal samples were found at a site, only one was collected to avoid over representing the site location. We classified fecal samples as fresh based on odor, consistency and appearance, and then collected them in sealed 50‐mL plastic vials. During the field seasons, scat samples were kept in cool, dark areas at our field camp and subsequently stored at −20°C in a lab at the end of each season. We then created composite samples by combining ~5‐mL of fecal material subsampled from three to five individual samples randomly selected for each year. This process was repeated 10 (for samples collected in 2018 and 2019) or 12 (for samples collected in 2023) times without replacement of fecal samples, and final composite samples were sent to Jonah Ventures (Boulder, USA) for diet content analysis using DNA metabarcoding (see Appendix S2a for details). Fecal samples collected in the core range before and after the herd migrated (i.e., between June 1st and July 15th) represented the winter and late summer diets, respectively. While samples collected in the neonatal range at the end of their migratory period, were considered as their late spring/early summer diet.

Composite samples were analyzed for plant DNA via sequencing of the chloroplast *trn*L intron a reliable approach to describe herbivore diets when short DNA fragments are present in degraded samples (Taberlet et al. 2007; Valentini et al. 2009; Craine et al. 2015) (see Appendix S2b for details). Sequences of DNA found in the samples were classified into operational taxonomic units (hereafter taxonomic units) by using the Basic Local Alignment Search Tool (BLAST) from the National Center for Biotechnology Information (Blaxter et al. 2005; NCBI 2023). BLAST identifies regions of similarity by comparing nucleotide sequences from samples with sequences of known organisms in its database (NCBI 2023). We grouped taxonomic units based on their similarity (> 97%) and their geographical distribution, considering only taxa that were known to be present in the area. When two or more species presented the same similarity for a single sequence and were known to be present in the area, we used the higher taxonomic level (i.e., genus or family), with the exception of sequences where *Rosa acicularis* and *Fragaria* spp. were present, where *Rosa acicularis* was given prevalence due to its dominance in the environment (see Appendix S2c for details). Using the number of times each sequence was read within each sample, we then calculated the relative read abundance (RRA) for each taxonomic unit as the read count of that unit divided by the total number of reads across all taxonomic units (Deagle et al. 2019; Hecker et al. 2021). RRA represents the percentage of DNA belonging to each taxonomic unit and is used as a reliable proxy of the relative consumption of each item (Deagle et al. 2019). Only taxonomic units that accounted for at least 1% of the diet were included, as this threshold helps to minimize the effect of low‐level background noise (i.e., low abundance sequences) (Deagle et al. 2019). Additionally, taxonomic units were categorized into four functional groups to identify dietary shifts throughout the year: graminoids (grasses and sedges), forbs (nongraminoid herbaceous plants), browse (coniferous and deciduous shrubs and trees), and an “other” category.

### 2.4 Forage Quantity and Quality Analyses

We quantified forage within each 0.0625‐m^2^ quadrat deployed at confirmed bison locations or original coordinates of selected sites. Within a three‐dimensional space above each quadrat, the foliar portion of all plant species was clipped from ground level to 2‐m above ground, the maximum foraging height for an adult bison. Individual samples were stored in breathable paper bags in a meshed enclosure to allow airflow and drying. Samples were later transported to a lab to be dried at 60°C for 24 h and weighed to measure dry biomass by species. Forage quantity was based on the dry biomass from plant species clipped inside each quadrat. To assess the forage quality, 20‐g samples from the most frequently found plant species at each bison range were analyzed for chemical nutritional content at Nutrilytical Lab (Calgary, Canada). Information regarding crude protein (mg/g) and metabolizable energy concentration (ME; kcal/g) was obtained for each species and an overall yield value for these nutritional components was then calculated for each quadrat by multiplying nutrient concentrations by dry matter biomass. Final dry biomass, crude protein yield, and ME yield values were expressed per square meter (m^2^) to facilitate comparisons between bison ranges.

Forage quantity and quality analyses were based only on plant species comprising at least 1% of the herd's diet throughout the year, and these species were grouped into one of three categories based on prevalent growth form: shrubs, forbs and graminoids. Additionally, only sites surveyed between 1 June and 15 July were included, because they represent the peak of vegetation green‐up in the area and coincide with the time when the herd is known to be in their neonatal range (see Appendix S2d,e for details). Due to the non‐normal distribution of data, we used nonparametric Mann–Whitney‐Wilcoxon tests to evaluate differences between the herd's neonatal and core range (i.e., our independent variable) for all forage groups, with medians and interquartile ranges (IQR) being presented. All data organization and analysis were performed using the software R 4.1.0 (R Core Team 2021).

## 3 Results

### 3.1 Seasonal Diet Content

We collected a total of 122 bison fecal samples. Of that total, 91 samples (74.6%) were from the core range representing the winter (premigration) and mid‐late summer (postmigration) diet, while 31 samples (25.4%) were from the neonatal range representing the late‐spring/early‐summer migration diet. The DNA analysis detected 386 unique sequence variants across all samples (seasons), but 73 less common variants were excluded due to being absent in the study area. This resulted in 58 unique taxonomic units for winter, 40 unique taxonomic units for late‐spring/early‐summer, and 59 unique taxonomic units for mid‐late summer with an overall cumulative read count of ~96%.

**FIGURE 2:**
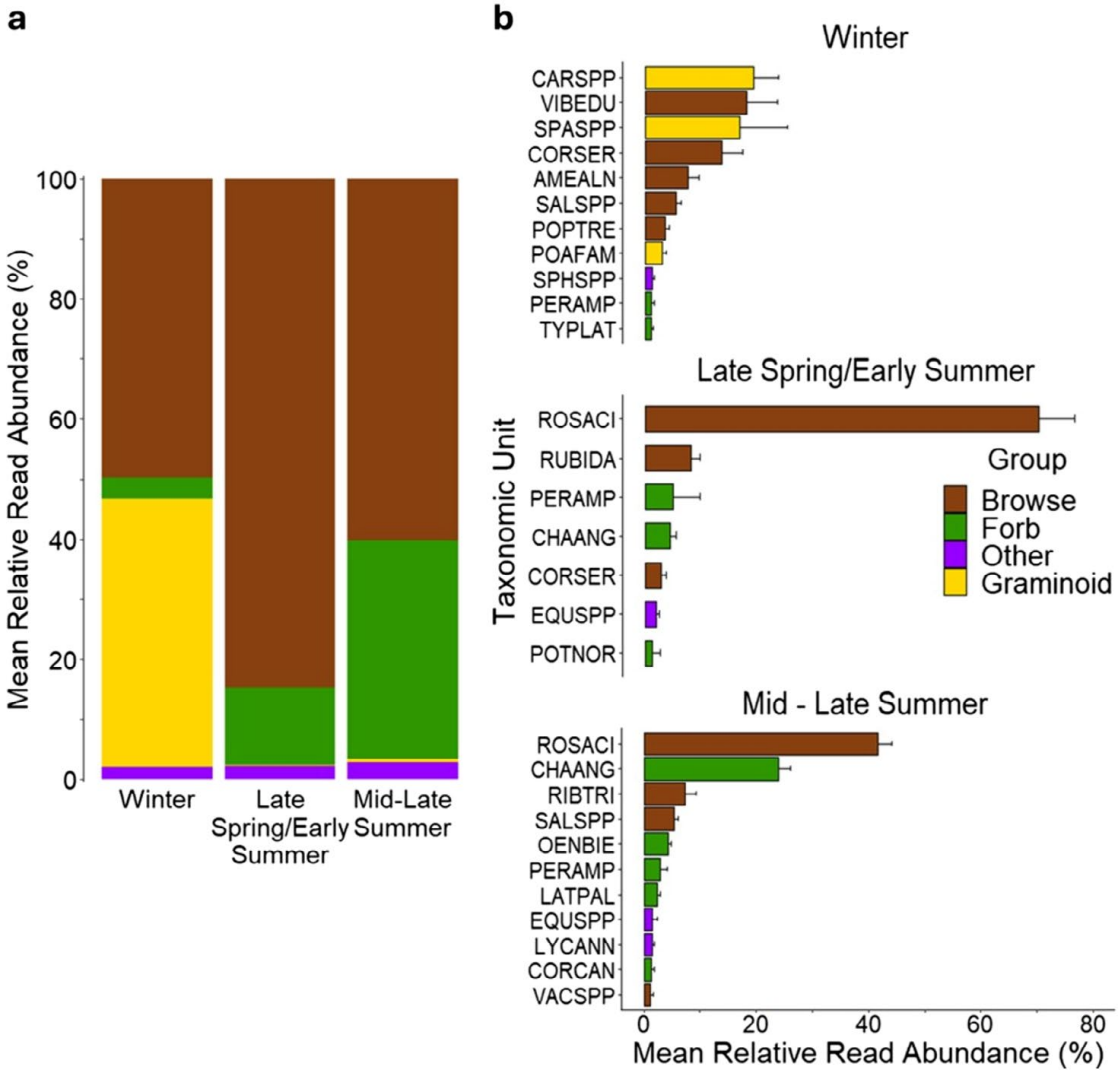
Seasonal dietary contribution of forage groups for the Ronald Lake Bison Herd in northeastern Alberta, Canada (a) and seasonal diets with taxonomic units that represent at least 1% of the diet identified as six‐letter species codes of family, genus, or species (see Table 1 and Appendix S2c for details) (b).

The herd's winter diet in the core range was composed of shrubby browse items (49.8%), followed closely by graminoids (44.6%), then forbs (3.5%), and other groups (2.1%). The three taxonomic units with the highest RRA values were *Carex* spp. (RRA = 19.6, SE = 4.5), *Viburnum edule* (RRA = 18.3, SE = 5.6), and *Sparganium* spp. (RRA = 17.1, SE = 8.7) (Figure 2b). The herd's late‐spring/early‐summer diet in the neonatal range was dominated by browse items (84.8%), followed by forbs (12.9%), and then other groups (2.2%) and graminoids (0.1%). Two shrub species, *Rosa acicularis* and *Rubus idaeus* , were the most prevalent, with an RRA of 71.2 (SE = 6.5) and 8.4 (SE = 1.5) respectively, followed by the forb *Persicaria amphibia* at 5.0 (SE = 4.9) (Figure 2b). The herd's mid‐late summer diet in the core range was dominated by browse (60.2%), followed by forbs (36.5%), other groups (2.8%) and graminoids (0.5%). *Rosa acicularis* had the highest RRA (RRA = 42.1, SE = 2.6), followed by *Chamaenerion angustifolium* (RRA = 24.3, SE = 2.2), and *Ribes triste* (RRA = 7.4, SE = 1.9) (Figure 2b; see Table 1 for details). Overall, we found strong seasonal changes in the herd's diet, going from a diet with abundant graminoids in winter to a shrub and forb‐dominated diet in late spring and through the summer (Figure 2a). It was notable that browse was the most dominant component of the herd's diet in their neonatal range during a period when browse items are exhibiting new spring growth.

**TABLE 1:** Identified taxonomic units for all seasons with their six‐letter species code used for RRA analysis.

Taxonomic unit	Family	Genus	Species	Forage group	Mean relative read abundance (RRA)		
					Winter	Late spring	Mid‐late summer
AMEALN	Rosaceae	*Amelanchier*	*alnifolia*	Browse	7.7	—	—
CARSPP	Cyperaceae	*Carex*	NA	Graminoid	19.6	—	—
CHAANG	Onagraceae	*Chamaenerion*	*angustifolium*	Forb	—	4.5	24.3
CORCAN	Cornaceae	*Cornus*	*canadensis*	Forb	—	—	1.3
CORSER	Cornaceae	*Cornus*	*sericea*	Browse	13.9	3.0	—
EQUSPP	Equisetaceae	*Equisetum*	NA	Other	—	2.1	1.5
LATPAL	Fabaceae	*Lathyrus*	*palustris*	Forb	—	—	2.4
LYCANN	Lycopodiaceae	*Lycopodium*	*annotinum*	Other	—	—	1.5
OENBIE	Onagraceae	*Oenothera*	*biennis*	Forb	—	—	4.4
PERAMP	Polygonaceae	*Persicaria*	*amphibia*	Forb	1.2	5.0	2.9
POAFAM	Poaceae	NA	NA	Graminoid	3.1	—	—
POTNOR	Rosaceae	*Potentilla*	*norvegica*	Forb	—	1.4	—
POPTRE	Salicaceae	*Populus*	*tremuloides*	Browse	3.7	—	—
RIBTRI	Grossulariaceae	*Ribes*	*triste*	Browse	—	—	7.4
ROSACI	Rosaceae	*Rosa*	*acicularis*	Browse	—	71.2	42.1
RUBIDA	Rosaceae	*Rubus*	*idaeus*	Browse	—	8.4	—
SALSPP	Salicaceae	*Salix*	NA	Browse	5.7	—	5.4
SPASPP	Sparganiaceae	*Sparganium*	NA	Graminoid	17.1	—	—
SPHSPP	Sphagnaceae	*Sphagnum*	NA	Other	1.4	—	—
TYPLAT	Typhaceae	*Typha*	*latifolia*	Graminoid	1.1	—	—
VACSPP	Ericaceae	*Vaccinium*	NA	Browse	—	—	1.2
VIBEDU	Caprifoliaceae	*Viburnum*	*edule*	Browse	18.3	—	—

*Note:* The symbol—indicates taxonomic units that constitute at least 1% of a seasonal diet but are not present in that specific season.

### 3.2 Forage Quantity

**FIGURE 3:**
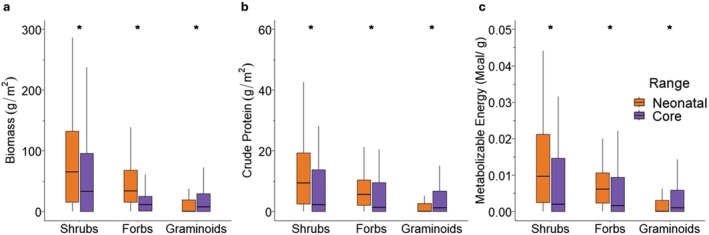
Comparison of dry biomass (a), crude protein yield (b), and metabolizable energy yield (c) between the neonatal and core ranges of the Ronald Lake Bison Herd in northeastern Alberta, Canada, for shrubs, forbs, and graminoids. The symbol * represents significant (*p* < 0.010) differences between the two ranges based on Wilcoxon tests.

Forage quantity was assessed across 348 sites (plots) between June 1 and July 15, of which 217 were in the core range and 131 in the neonatal range (see Appendix S3 for detailed distribution of plots). The neonatal range had significantly higher biomass of shrubs (*p* ≤ 0.010) and forbs (*p* ≤ 0.001), with median values of 71.20‐g/m^2^ (IQR = 16.08–140.40) for shrubs and 41.04‐g/m^2^ (IQR = 15.44–70.48) for forbs. Conversely, graminoids were significantly more abundant (*p* ≤ 0.001) in the core range at a median dry biomass of 8.80‐ g/m^2^ (IQR = 0–34.88), approximately 18 times greater than in the neonatal range. Shrub and forb biomass were 1.7 and 3.8 times higher, respectively, in the neonatal range compared with the core range (Figure 3a; details can be found in Appendix S4).

### 3.3 Forage Quality

We quantified forage quality from 228 sites between June 1 and July 15, of which 97 were from the core range and 131 were from the neonatal range (see Appendix S5 for detailed distribution of plots). Crude protein yields were significantly higher for shrubs (*p* < 0.001) and forbs (p < 0.001) in the neonatal range compared with the core range, being 2.7 and 3.2 times higher for shrubs (median = 10.11 g/m^2^, IQR = 2.71–23.41) and forbs (median = 6.05 g/m^2^, IQR = 2.15–12.43), respectively (Figure 3b). The core range had the highest crude protein yield for graminoids (*p* < 0.001), being ~46 times higher than the neonatal range (median = 1.53 g/m^2^, IQR = 0–7.01). The neonatal range also had a greater ME yield of shrubs (*p* < 0.001) and forbs (p < 0.001), being 3.0 (median = 170.64 (kcal/g)/m^2^, IQR = 41.39–365.46) and 3.7 (median = 105.65 (kcal/g)/m^2^, IQR = 38.37–190.15) times higher, respectively. However, the core range had a higher ME yield for graminoids (*p* < 0.005), being 36 times higher than the neonatal range (median = 22.18 (kcal/g)/m^2^, IQR = 0–102.78) (Figure 3c; details can be found in Appendix S6).

## 4 Discussion

Our findings reveal a clear shift in the herd's diet between the ranges, as hypothesized. During winter in the core range, the herd consumed primarily graminoids and shrubs. However, in late spring and early summer within the neonatal range, their diet shifted to predominantly shrubs. By mid‐to‐late summer, as bison returned to their core range, their diet was more balanced between shrubs and forbs (Figure 2a). We suggest that the shift in seasonal diets was due to ongoing changes in temporal and spatial availability of forage, and importantly, the ability of bison to migrate and opportunistically exploit increased resources (i.e., crude protein and energy yield) within the neonatal range. In winter, when graminoids constituted a larger component of the herd's diet, forage options were limited due to the absence of green foliage. Forbs are known for being low in availability and quality after the growing season (Balde et al. 1993; Buxton and Fales 1994), and while shrubs may provide ample biomass, the latter is typically lower in abundance due to leaf‐fall, and ongoing lignification of new stems leads to low forage quality (Buxton and Fales 1994; Cook et al. 2016). After snowmelt and the onset of spring, the new growth of shrubs and forbs became available for bison (Hartley and Jones 1996). The composition of available forage also differed between ranges, with the core range containing numerous wetlands that are rich in graminoid forage, while the neonatal range is dominated by upland ecosystems that are much lower in abundance of graminoids.

Bison have traditionally been considered as primarily grazers, meaning that their diets are dominated by grasses and sedges, with forbs and shrubs being minimal or absent in their diets (Peden et al. 1974; Coppedge et al. 1998; Shipley 1999). Previous studies have documented a strong summer preference for graminoids, with microhistological analysis reporting up to 99% of graminoid consumption by plains bison ( *Bison bison bison* ) in the Great Plains of the United States (Coppedge et al. 1998; Fortin et al. 2003). These findings contrast sharply with our results. We observed dietary plasticity between successive seasons of growth, indicative of changes in foraging behavior, with bison transitioning from a grazing‐dominated behavior in winter, to a browsing‐dominated behavior in spring and summer. This seasonal shift aligns with growing evidence that bison foraging strategies are more flexible than previously assumed, particularly in response to seasonal changes in forage availability and quality (Bergmann et al. 2015; Leonard et al. 2017; Craine 2021; Hecker et al. 2021). Discrepancies with earlier studies could be partially explained by biases in diet assessment, as well as differences in focal subspecies and study locations, with most early work taking place on plains bison in grassland dominated landscapes of the United States.

Our results also supported our hypothesis regarding forage quantity and quality differences between bison ranges, particularly that of shrubs and forbs. The neonatal range presented the highest values of available biomass, crude protein yield, and ME yield for shrubs and forbs, while biomass, crude protein, and ME yields for graminoids were higher in the core range. These discrepancies in the quantity and quality of forage can be attributed to the different ecosystems between ranges. The neonatal range is predominantly composed of upland deciduous forest and upland meadows, which are rich in forbs and shrubs, whereas the core range is characterized by forest and wetland ecosystems, with the wetlands abundant in graminoid vegetation. Although graminoid forage is rich in fiber, it contains lower concentrations of protein and energy during the growing season compared to shrubs and forbs (Lee 2018). The neonatal range also features an extensive and continuous upland shrubby meadow used extensively by bison during this period, unique in the herd's range, that is dominated by the plant species prickly rose and raspberry, which were the two most abundant species quantified in the herd's diet while in the neonatal range. Prickly rose also emerged as the primary dietary species in the core range during mid and late summer (Figure 2b). These species are of high nutritional value, with prickly rose presenting the highest ME concentration (2.78 Mcal/kg) and raspberry ranking among the top three species in crude protein content (19.8%) among all shrub species evaluated in the neonatal range (Appendix S7).

It is no surprise that the bison range with higher biomass for a specific forage type, also showed higher crude protein and ME yields within the forage types, as nutrient yield is a combined function of biomass and nutrient concentrations. However, we also found that the magnitude of the differences between ranges in quality was not always explained directly by forage quantity alone. For instance, while shrub biomass in the neonatal range was 1.7 times greater than in the core range, shrub crude protein and ME yields were 2.7 and 3.0 times higher, respectively, than in the core range (Appendices S4 and S6). This highlights the key benefit of increases in forage quality in contributing to overall nutrient increases for bison within the neonatal range. In contrast, forb biomass in the neonatal range was 3.8 times greater, which paralleled its increases in crude protein and ME yield of 3.2 and 3.7, respectively, compared with the core range, indicating that the primary benefit from forbs in the neonatal range may have been an increase in abundance. Given that ME and crude protein are critical for calf growth and overwinter survival (Cook et al. 2004; Tollefson et al. 2011), these results suggest that forage quality, particularly of shrubs, plays a more crucial role than quantity in the herd's migration during their neonatal period. Our results support previous studies reporting that herbivore migrations are primarily influenced by forage quality, especially when animals are under energetic stress (Hebblewhite et al. 2008; Cagnacci et al. 2011; Merkle et al. 2016).

Tracking the phenological waves of highly nutritious new forage, a concept coined as the “greenwave hypothesis,” has received empirical support as being one of the best explanations for the timing and extent of migratory movements in ungulates (Van Soest 1982; Bischof et al. 2012; Aikens et al. 2017). However, various responses in how animals track new forage have been documented, with some populations surfing the greenwave as it advances over time (Aikens et al. 2017; Sigrist et al. 2022), others jumping it and waiting for it to arrive at their final summer range (Bischof et al. 2012; Laforge et al. 2021), and in some cases, ungulates may even manipulate the greenwave through intense foraging (Geremia et al. 2019). We did not directly evaluate the strategy that the herd uses to track new growth, but a previous study found that it is unlikely that the herd is surfing the greenwave (Hecker 2022). The spatial variation in phenology associated with terrain and landform differences between the core and neonatal range is minimal due to its low relief, and the 28‐km distance traveled between ranges is not far enough to substantially alter plant phenology. Further studies are needed to better understand how the herd tracks the new growth to maximize their energy intake. As green‐up occurs at approximately the same time between the core and neonatal range, this suggests it has less to do with phenology than differences in the quantity and quality of available forage.

Our study focused on the influence of forage characteristics on the herd's migration, but other factors may also play an important role in explaining bison movements. In many cases, ungulates migrate to seasonal ranges to reduce predation risk for themselves or their calves (Festa‐Bianchet 1988; Fryxell and Sinclair 1988; Hebblewhite and Merrill 2007). Consequently, predator pressure could be influencing the herd's migration. Wolves and black bears ( *Ursus americanus* ) are the only species within their range that could prey on bison or their calves. Wolves, considered the primary predators of bison in North America, can exert top‐down control on bison populations, including those in Wood Buffalo National Park just north of Ronald Lake (Joly and Messier 2004). Although black bears are not the main predators of bison due to their size and omnivorous diet, there is evidence that they opportunistically prey on other ungulates and their calves (Bowersock et al. 2021; Bonin et al. 2023). Previous studies on wolves and black bears have shown limited predation pressure on the herd (Dewart 2023; Sharp et al. 2025), although predators could influence the herd's migration indirectly. By aggregating in larger numbers or moving to areas where predation risk is perceived to be lower, individuals can spend less time being vigilant and more time foraging (Ximming et al. 2007; Christianson and Creel 2010). This is crucial for the herd, as females need to meet their higher nutritional requirements while simultaneously protecting their neonates from possible predators.

Insect harassment is another possible factor influencing the herd's migration. Biting insects have been shown to directly affect ungulate foraging behaviors and habitat selection, including that of bison (Hagemoen and Reimers 2002; Witter et al. 2012; Belanger et al. 2020). Insect harassment can have adverse fitness consequences due to the increased nutritional demands associated with reduced food intake and the increase of avoidance behaviors (Fitze et al. 2004; Benedict and Barboza 2022; Johnson et al. 2022). This is especially true for calves, as evidence shows that insect harassment can affect their weight and survival (Weladgi et al. 2003; Johnson et al. 2022). Some of the most common ectoparasitic insects in boreal forests, including the families Simuliidae, Culicidae, and Tabanidae, use wetlands as breeding grounds (Lewis 1987), which are prevalent in the core range of the herd. Thus, insect harassment may be one component explaining the herd's movements to the neonatal range, further supported by our findings that their diet is dominated by graminoids during winter, when insect harassment is absent, and its presence in the diet decreases during spring and summer (Figure 2a).

Apart from the factors not considered here that could also influence the herd's migration and diet, we acknowledge that our study also presents some limitations. Our diet results come from a DNA metabarcoding approach, which infers diet from the proportion of sequence reads recovered from fecal material. This semi‐quantitative method can introduce bias due to the differential digestion of food taxa and DNA degradation, which may not always accurately reflect the actual proportion of consumed plants (Nakahara et al. 2015; Deagle et al. 2019). Taxonomic identification further relies on the comparison of an amplified DNA fragment (the chloroplast *trnL* intron in our case) to a reference database; however, global barcode coverage is still incomplete, meaning that some of our locally occurring species may go undetected or may be assigned to the wrong taxon (Pompanon et al. 2012). This limitation could explain the relatively high number of sequence variants (i.e., 73) that could not be assigned to known plant species in the area using the BLAST database (NCBI 2023). Additionally, we used a 1% RRA threshold to filter the identified taxa, which, despite being frequently used in the literature (McInnes et al. 2017; Deagle et al. 2019; Hecker et al. 2021), could exclude important food items and modify ecological interpretations (Littleford‐Colquhoun et al. 2022). Nevertheless, we ran our diet analyses without applying any threshold, and we did not find any alteration in the relative order of functional groups for each season, with very similar proportional contributions for each category (see Appendix S8 for details).

Furthermore, it is important to note that our forage quantity and quality estimates are based on data from sites visited after they were utilized by bison, leading to an underestimation of the actual availability at the time of utilization. Additionally, our findings may also be influenced by the effect of foraging on vegetation regrowth. Browsed shrubs and forbs can exhibit lower quality compared to unbrowsed individuals, while grazed graminoids undergoing regrowth may offer higher nutritional value than their ungrazed counterparts (Raynor et al. 2016). These effects of browsing and grazing on forage quality likely amplify the differences already observed between the neonatal and core range.

## 5 Conclusions

Our study provides valuable insights into the factors influencing the migration of the RLBH. We found a clear shift in the herd's diet between its core (winter) and neonatal ranges (spring/early summer), with a heavily browse‐dominated diet in late spring and early summer when the herd occupies the neonatal range with their young calves. Our results suggest that this dietary shift in the neonatal range is influenced by the greater abundance and relative quality of forbs, and especially shrubs, that this range offers in comparison to the core range, with forage quality (i.e., nutrient yield) possibly playing an even more significant role than quantity in the selection of this area during their neonatal period. While other factors may influence the herd's migration, our results suggest a link between forage quantity and quality with bison migration in late spring, which aligns with the higher nutritional requirements that female bison are experiencing during this time of the year. Ultimately, this study may help identify and understand critical habitat for bison during a period of the year when they are nutritionally limited and provides a foundation for future work aiming to quantify forage–habitat relationships in the area This is particularly important for this herd, as its range is close to areas of oil sand exploration and development and its conservation has significant ecological and cultural implications.

## Author Contributions

**Sebastian Buitrago Gutierrez:** conceptualization (lead), data curation (lead), formal analysis (lead), funding acquisition (supporting), investigation (lead), methodology (lead), validation (lead), visualization (lead), writing – original draft (lead), writing – review and editing (lead). **Lee J. Hecker:** data curation (supporting), investigation (supporting), writing – review and editing (equal). **Edward W. Bork:** conceptualization (equal), methodology (supporting), supervision (supporting), writing – review and editing (equal). **Mark A. Edwards:** conceptualization (equal), funding acquisition (lead), investigation (supporting), methodology (equal), project administration (lead), resources (lead), supervision (equal), visualization (supporting), writing – review and editing (equal). **Scott E. Nielsen:** conceptualization (equal), formal analysis (supporting), funding acquisition (lead), investigation (supporting), methodology (equal), project administration (lead), resources (lead), supervision (lead), validation (supporting), visualization (equal), writing – review and editing (equal).

## Funding

Funding was provided by: Government of Alberta, Fish and Wildlife, Alberta Conservation Association: Grants in Biodiversity, and University of Alberta Northern Research Awards – Northern Scientific Training Program.

## Conflicts of Interest

The authors declare no conflicts of interest.

## Supporting information


**Appendix S1:** Sampling process and field photographs in the Ronald Lake wood bison herd ranges. Circular quadrat (0.0625‐m2) used to clip vegetation (a); field technician collecting information in a wallow area in the neonatal range (b); on‐site storage of clipped vegetation samples (c); graminoid wetland in the core range (d); shrubby meadow complex in the neonatal range (e); summer aerial view of the core range (f); summer aerial view of the meadow complex in the neonatal range (g); and fall aerial view of the meadow complex in the neonatal range (h).Photographs a, c, e, and f were taken by Ivy Boddez; b was taken by Garrett Rawleigh; d was taken by Darren Epperson; g was taken by Amber Harris; and h was taken by Scott Nielsen.

**Appendix S2:** Detailed methods for composite samples creation (a), DNA metabarcoding (b), seasonal diet content (c), and forage quantity (d) and quality (e) analyses.

**Appendix S3:** Distribution of sites sampled for biomass analyses in the Ronald Lake wood bison herd ranges (a), with a more detailed view of the 131 sites in the neonatal range (b) and the 217 sites in the core range (c) shown.

**Appendix S4:** Biomass median values, interquartile ranges (IQR), and Wilcoxon test *p* values between ranges and forage groups.

**Appendix S5:** Distribution of sites sampled for macronutrient analysis in the Ronald Lake wood bison herd ranges (a), with a more detailed view of the 131 sites in the neonatal range (b) and the 97 sites in the core range (c) shown.

**Appendix S6:** Protein and ME median yield values, interquartile ranges (IQR), and Wilcoxon test *p* values comparing bison ranges withing each of the forage groups.

**Appendix S7:** Protein and ME raw values for the most frequent plant species in the neonatal range during early summer, listed by growth form and in decreasing quality.

**Appendix S8:** Seasonal dietary contribution percents of forage groups for the Ronald Lake Wood Bison Herd, including all identified taxa with no exclusion threshold applied (i.e., taxa that accounted for at least 1% of the diet).

## Data Availability

Data supporting the findings of this study can be found on Dryad: DOI: https://doi.org/10.5061/dryad.wpzgmsc1p.
