# Supplementary material for: The Role of Forage Quantity and Quality in the Migration and Diet of a Northern Ungulate During Their Neonatal Period
Source: Ecol Evol. 2026 Apr 8;16(4):e73454. doi: 10.1002/ece3.73454 (PMC13062649; doi:10.1002/ece3.73454)
Supplement: Supplementary file 1 — Appendix S1: Sampling process and field photographs in the Ronald Lake wood bison herd ranges. Circular quadrat (0.0625‐m2) used to clip vegetation (a); field technician collecting information in a wallow area in the neonatal range (b); on‐site storage of clipped vegetation samples (c); graminoid wetland in the core range (d); shrubby meadow complex in the neonatal range (e); summer aerial view of the core range (f); summer aerial view of the meadow complex in the neonatal range (g); and fall aerial view of the meadow complex in the neonatal range (h).Photographs a, c, e, and f were taken by Ivy Boddez; b was taken by Garrett Rawleigh; d was taken by Darren Epperson; g was taken by Amber Harris; and h was taken by Scott Nielsen. [file ECE3-16-e73454-s005.pdf]

Appendix 1. Sampling process and field photos in the Ronald Lake wood bison herd ranges. Circular quadrat (0.0625-m^2) used to clip vegetation (a); field technician collecting information in a wallow area in the neonatal range (b); on-site storage of clipped vegetation samples (c); graminoid wetland in the core range (d); shrubby meadow complex in the neonatal range (e); summer aerial view of the core range (f); summer aerial view of the meadow complex in the neonatal range (g); and fall aerial view of the meadow complex in the neonatal range (h).Photographs a, c, e, and f were taken by Ivy Boddez; b was taken by Garrett Rawleigh; d was taken by Darren Epperson; g was taken by Amber Harris; and h was taken by Scott Nielsen.

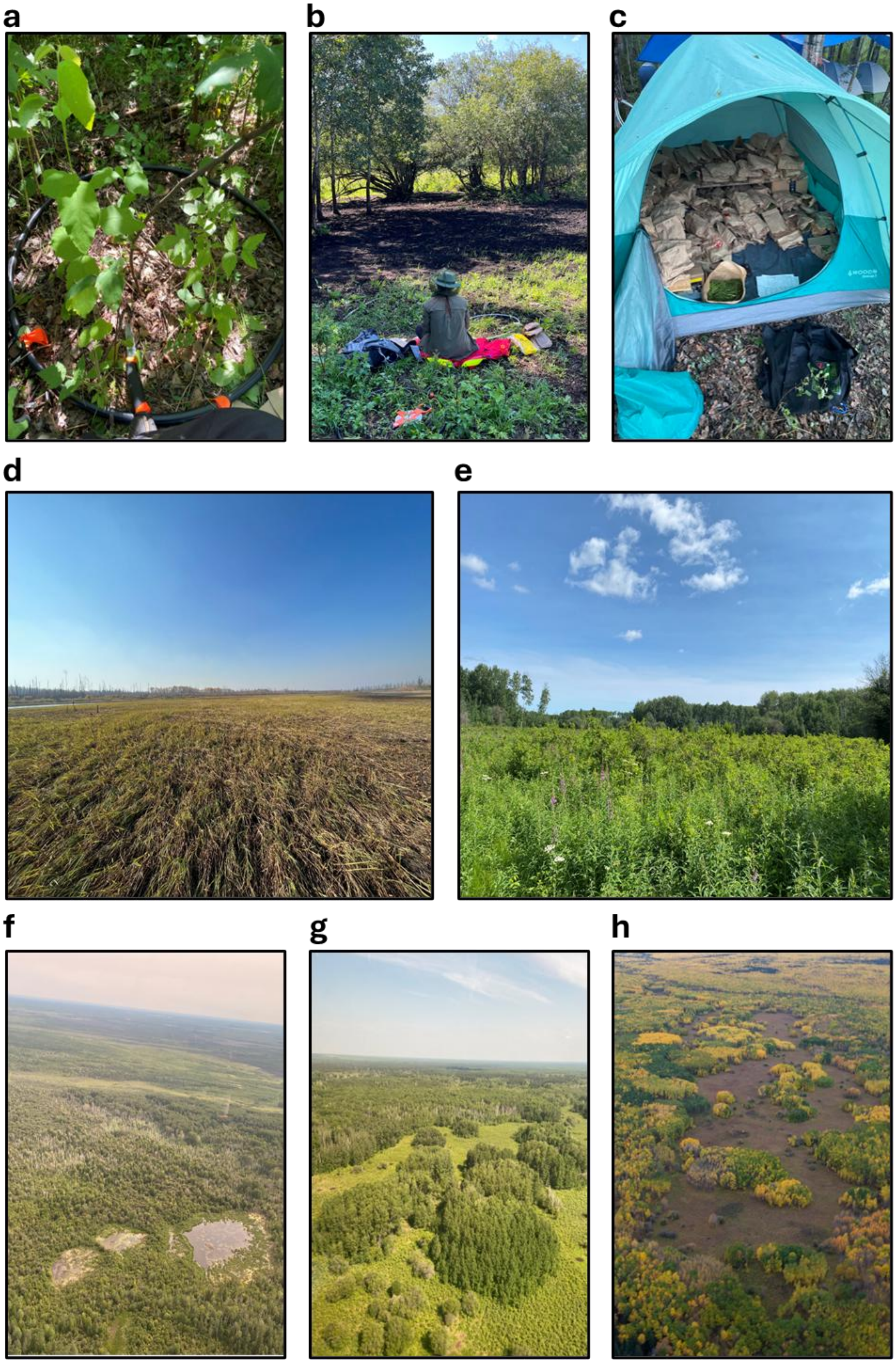
a
b
c
d
e
f
g
h
