# Supplementary material for: The Role of Forage Quantity and Quality in the Migration and Diet of a Northern Ungulate During Their Neonatal Period
Source: Ecol Evol. 2026 Apr 8;16(4):e73454. doi: 10.1002/ece3.73454 (PMC13062649; doi:10.1002/ece3.73454)
Supplement: Supplementary file 2 — Appendix S2: Detailed methods for composite samples creation (a), DNA metabarcoding (b), seasonal diet content (c), and forage quantity (d) and quality (e) analyses. [file ECE3-16-e73454-s007.pdf]

Appendix 1. Detailed methods for composite samples creation (a), DNA metabarcoding (b), seasonal diet content (c), and forage quantity (d) and quality (e) analyses.

a. If multiple samples were found around a site, only one fresh sample was collected to avoid over representing a location or individual. In the core range, scat samples were collected only around sites of known bison locations that were visited within 10-days of the bison presence. Each site was visited only one time. Composite samples were created by randomly selecting three to five individual fecal samples per season from the same year, and this process was repeated 10 times for winter and late summer seasons in the core range, and 12 times for late spring/early summer in the neonatal range. Composite samples were first created for the scat collected in 2018 and 2019, and the same process was applied to the 2023 samples to maintain consistency in the design.

b. Genomic DNA from samples was extracted using the DNeasy 96 PowerSoil Pro Kit (384) (Cat # 47017) according to the manufacturer's protocol. Genomic DNA was eluted into 100µl and frozen at -20°C. A portion of the chloroplast trnL intron was PCR amplified from each genomic DNA sample using the c and h trnL primers. Both forward (i.e., CGAAATCGGTAGACGCTACG) and reverse (i.e., CCATTGAGTCTCTGCACCTATC) primers also contained a 5' adaptor sequence to allow for subsequent indexing and Illumina sequencing. Each 25 µL PCR reaction was mixed according to the Promega PCR Master Mix specifications (Promega catalog # M5133, Madison, WI) which included 0.4 µM of each primer and 1 µl of gDNA. DNA was PCR amplified using the following conditions: initial denaturation at 94 °C for 3 minutes, followed by 40 cycles of 30 seconds at 94 °C, 30 seconds at 55 °C, and 1 minute at 72 °C, and a final elongation at 72° C for 10 minutes.

To determine amplicon size and PCR efficiency, each reaction was visually inspected using a 2% agarose gel with 5µl of each sample as input. Amplicons were then cleaned by incubating

amplicons with Exo1/SAP for 30 minutes at 37C following by inactivation at 95C for 5 minutes and stored at -20C.

A second round of PCR was performed to complete the sequencing library construct, appending with the final Illumina sequencing adapters and integrating a sample-specific,12-nucleotide index sequence. The indexing PCR included Promega Master mix, 0.5 µM of each primer and 2 µl of template DNA (cleaned amplicon from the first PCR reaction) and consisted of an initial denaturation of 95 °C for 3 minutes followed by 8 cycles of 95 °C for 30 sec, 55 °C for 30 seconds and 72 °C for 30 seconds.

Final indexed amplicons from each sample were cleaned and normalized using SequalPrep Normalization Plates (Life Technologies, Carlsbad, CA). 25µl of PCR amplicon is purified and normalize using the Life Technologies SequalPrep Normalization kit (cat#A10510-01) according to the manufacturer's protocol. Samples are then pooled together by adding 5µl of each normalized sample to the pool. Sample library pools were sent for sequencing on an Illumina MiSeq (San Diego, CA) at the Texas A&M Agrilife Genomics and Bioinformatics Sequencing Core facility using the v2 500-cycle kit (cat# MS-102-2003). Necessary quality control measures were performed at the sequencing center prior to sequencing.

Sample library pools were sent for sequencing on an Illumina MiSeq (San Diego, CA) at the Texas A&M Agrilife Genomics and Bioinformatics Sequencing Core facility using the v2 500-cycle kit (cat# MS-102-2003). Necessary quality control measures were performed at the sequencing center prior to sequencing.

Raw sequence data were demultiplexed using pheniqs v2.1.0, enforcing strict matching of sample barcode indices (i.e, no errors). Cutadapt v3.4 was then used remove gene primers from

the forward and reverse reads, discarding any read pairs where one or both primers were not found at the expected location (5') with an error rate < 0.15. Read pairs were then merged using vsearch v2.15.2, discarding resulting sequences with a length of < 100 bp or with a maximum expected error rate > 0.5 bp. For each sample, reads were then clustered using the unoise3 denoising algorithm as implemented in vsearch, using an alpha value of 5 and discarding unique raw sequences observed less than 8 times. Counts of the resulting exact sequence variants (ESVs) were then compiled and putative chimeras were removed using the uchime3 algorithm, as implemented in vsearch. For each final ESV, a consensus taxonomy was assigned using a custom best-hits algorithm and a reference database consisting of publicly available sequences (GenBank) as well as Jonah Ventures voucher sequences records. Reference database searching used an exhaustive semi-global pairwise alignment with vsearch, and match quality was quantified using a custom, query-centric approach, where the % match ignores terminal gaps in the target sequence, but not the query sequence. The consensus taxonomy was then generated using either all 100% matching reference sequences or all reference sequences within 1% of the top match, accepting the reference taxonomy for any taxonomic level with > 90% agreement across the top hits.

c. Taxonomic units' assignment of unique sequence variants found in the scat composite samples was based on the known distribution of plant species. This distribution was determined based on the book "Plants of the Western Forest: Alberta, Saskatchewan & Manitoba. Boreal and Aspen Parkland" (Johnson et al., 2020) and public from the iNaturalist database (https://www.inaturalist.org/). Only plant species documented to be present in the boreal forest in the northern part of the province were considered for taxonomic unit's assignment.

When two or more species presented the same percent of similarity for a single sequence and were known to be present in the area, the higher taxonomic unit (i.e., genus or family) was used. However, an exception was made for some sequences found in composite samples representing the late-spring/early-summer diet in the neonatal range. These sequences accounted for 199550 number of reads (~72% of the total reads for that season) but could not differentiate between the species prickly rose (*Rosa acicularis*), woodland strawberry (*Fragaria vesca*), and virginia strawberry (*Fragaria virginiana*), all of which are present in the area but vary dramatically in their abundance (*Fragaris spp.* being less common). Ultimately, these sequences were assigned to *Rosa acicularis* (ROSACI). Field data indicated that prickly rose was the most prevalent between these species in the neonatal range, being found in ~79% of sampled sites, with a collected biomass of 529.9-g. In contrast, the combined presence of strawberry species (which would not be distinguished from each other in the field) was found in only ~12% of plots, with a collected biomass of 14.0-g.

d. Biomass values were based on clipped vegetation of the foliar portion for all plant species found in each quadrat. However, only species comprising at least 1% of the herd's diet in any season, based on seasonal diet content results, were included in the analysis. Moss species (i.e. *Sphagnum* spp.) were excluded from biomass estimation. The total biomass for each plant species was calculated per quadrat and then categorized into shrubs, forbs, and graminoids. Each quadrat had an area of 0.0625-m^2, hence all biomass values were divided by 0.0625 to represent values in g/m^2.

e. Macronutrient (i.e., protein and ME) analysis followed the same steps taken for the biomass analysis. Protein values were originally obtained as the percentage of the total dry biomass per plant species, it was necessary to multiply this percent by the biomass of each species per

quadrat to determine the total protein content. ME values, originally expressed in Mcal/kg (i.e., Megacalories per kilogram), were divided by 1000 and then multiplied by the total biomass of each plant species per quadrat to obtain a final value of Mcal/g (i.e., Megacalories per gram).
