# Supplementary material for: The Role of Forage Quantity and Quality in the Migration and Diet of a Northern Ungulate During Their Neonatal Period
Source: Ecol Evol. 2026 Apr 8;16(4):e73454. doi: 10.1002/ece3.73454 (PMC13062649; doi:10.1002/ece3.73454)

Appendix 3. Distribution of sites sampled for biomass analyses in the Ronald Lake wood bison herd ranges (a), with a more detailed view of the 131 sites in the neonatal range (b) and the 217 sites in the core range (c) shown.

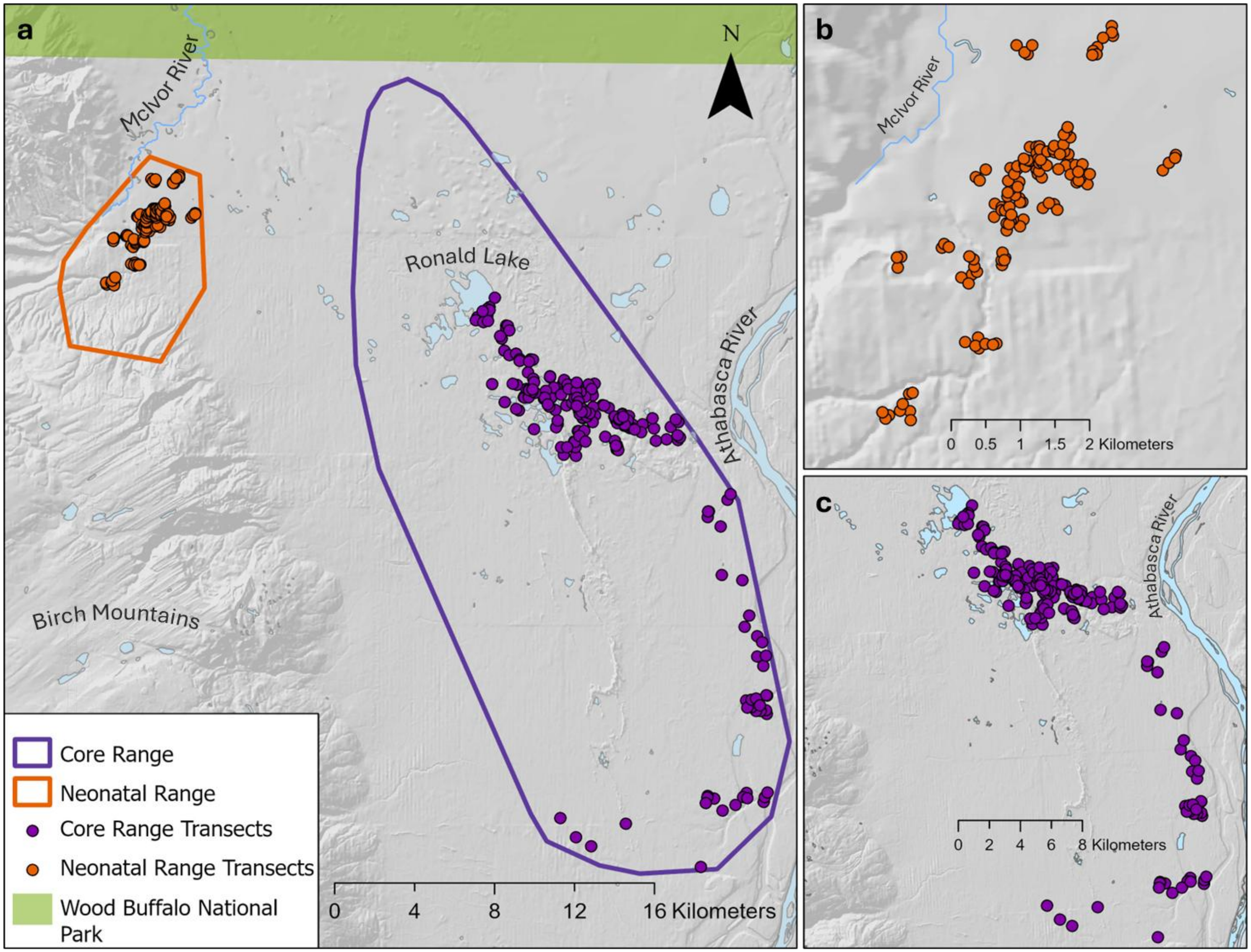

Supplement: Supplementary file 3 — Appendix S3: Distribution of sites sampled for biomass analyses in the Ronald Lake wood bison herd ranges (a), with a more detailed view of the 131 sites in the neonatal range (b) and the 217 sites in the core range (c) shown. [file ECE3-16-e73454-s003.pdf]
