# Supplementary material for: The Role of Forage Quantity and Quality in the Migration and Diet of a Northern Ungulate During Their Neonatal Period
Source: Ecol Evol. 2026 Apr 8;16(4):e73454. doi: 10.1002/ece3.73454 (PMC13062649; doi:10.1002/ece3.73454)
Supplement: Supplementary file 4 — Appendix S4: Biomass median values, interquartile ranges (IQR), and Wilcoxon test p values between ranges and forage groups. [file ECE3-16-e73454-s001.pdf]

Appendix 4. Biomass median values, interquartile ranges (IQR), and Wilcoxon test p-values between ranges and forage groups.

Forage item	Range	Biomass (g/m^2)	IQR	P-value
Shrubs	Core	41.12	0-124.48	≤ 0.010
	Neonatal	71.20	16.08-140.40	
Forbs	Core	10.88	0.96-25.12	≤ 0.001
	Neonatal	41.04	15.44-70.48	
Graminoids	Core	8.80	0-34.88	≤ 0.001
	Neonatal	0.50	0-19.60	
