# Supplementary material for: The Role of Forage Quantity and Quality in the Migration and Diet of a Northern Ungulate During Their Neonatal Period
Source: Ecol Evol. 2026 Apr 8;16(4):e73454. doi: 10.1002/ece3.73454 (PMC13062649; doi:10.1002/ece3.73454)

**Appendix 5.** Distribution of sites sampled for macronutrient analysis in the Ronald Lake wood bison herd ranges (a), with a more detailed view of the 131 sites in the neonatal range (b) and the 97 sites in the core range (c) shown.

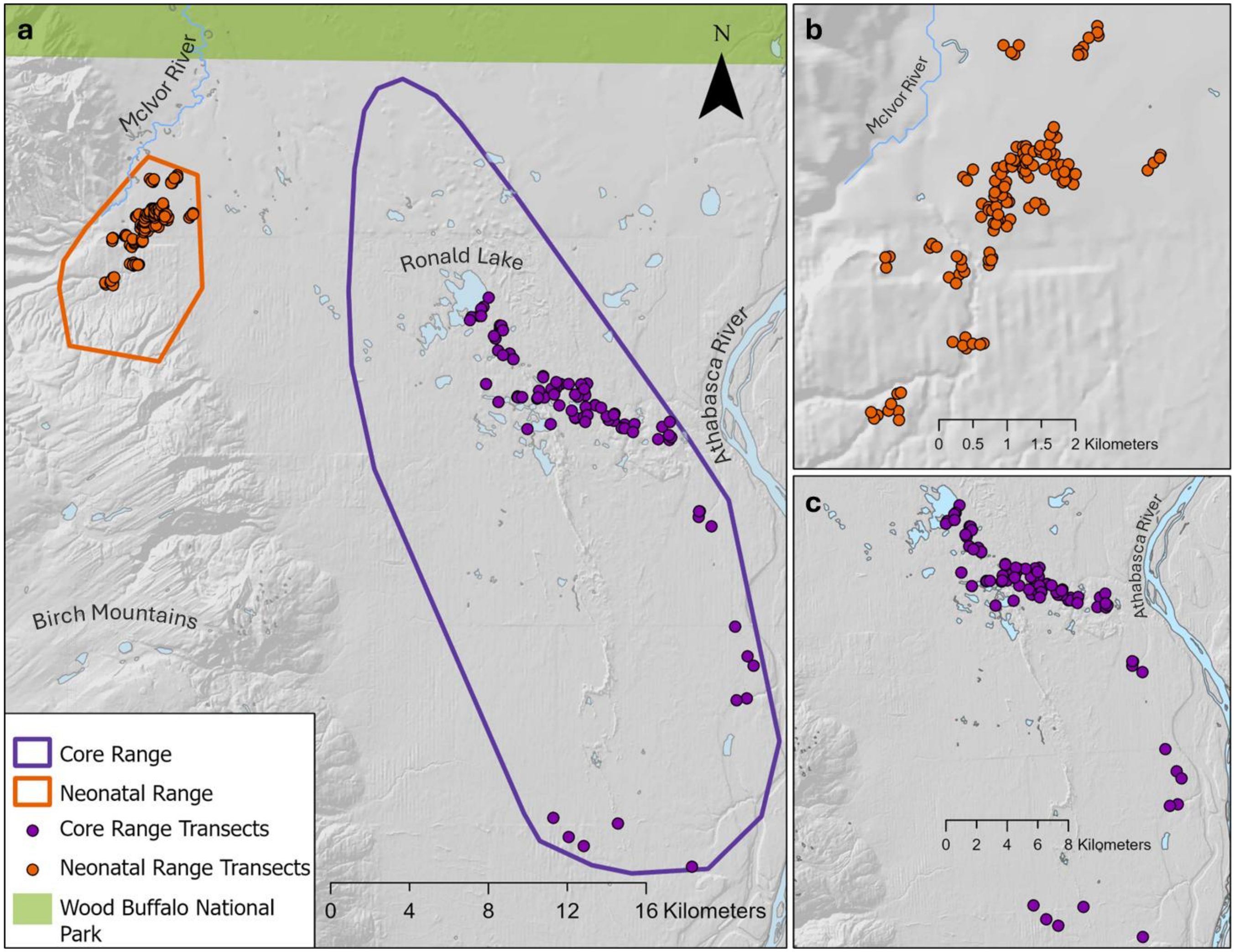

Supplement: Supplementary file 5 — Appendix S5: Distribution of sites sampled for macronutrient analysis in the Ronald Lake wood bison herd ranges (a), with a more detailed view of the 131 sites in the neonatal range (b) and the 97 sites in the core range (c) shown. [file ECE3-16-e73454-s004.pdf]
