# Supplementary material for: The Role of Forage Quantity and Quality in the Migration and Diet of a Northern Ungulate During Their Neonatal Period
Source: Ecol Evol. 2026 Apr 8;16(4):e73454. doi: 10.1002/ece3.73454 (PMC13062649; doi:10.1002/ece3.73454)
Supplement: Supplementary file 6 — Appendix S6: Protein and ME median yield values, interquartile ranges (IQR), and Wilcoxon test p values comparing bison ranges withing each of the forage groups. [file ECE3-16-e73454-s008.pdf]

Appendix 6. Protein and ME median yield values, interquartile ranges (IQR), and Wilcoxon test p-values comparing bison ranges withing each of the forage groups.

Forage item	Range	Protein (g/m^2)	IQR	P-value	ME ((kcal/g)/m^2)	IQR	P-value
Shrubs	Core	3.77	0-18.39	≤ 0.001	57.21	0-306.91	≤ 0.001
	Neonatal	10.11	2.71–23.41		170.64	41.39-365.46	
Forbs	Core	1.87	0–10.23	≤ 0.001	28.44	0-157.71	≤ 0.001
	Neonatal	6.05	2.15-12.43		105.65	38.37-190.15	
Graminoids	Core	1.53	0–7.01	≤ 0.001	22.18	0-102.78	≤ 0.005
	Neonatal	0.03	0-2.69		0.61	0-51.61	
