# Supplementary material for: The Role of Forage Quantity and Quality in the Migration and Diet of a Northern Ungulate During Their Neonatal Period
Source: Ecol Evol. 2026 Apr 8;16(4):e73454. doi: 10.1002/ece3.73454 (PMC13062649; doi:10.1002/ece3.73454)
Supplement: Supplementary file 7 — Appendix S7: Protein and ME raw values for the most frequent plant species in the neonatal range during early summer, listed by growth form and in decreasing quality. [file ECE3-16-e73454-s006.pdf]

Appendix 7. Protein and ME raw values for the most frequent plant species in the neonatal range during early summer, listed by growth form and in decreasing quality.

Forage item	Plant species	Protein (%)	ME (kcal/kg)
Shrub	*Ribes* spp.	20.7	2200
Shrub	*Rubus idaeus*	19.8	2600
Shrub	*Aralia nudicaulis*	15.1	2100
Shrub	*Prunus* spp.	15.1	2100
Shrub	*Viburnum opulus*	14.0	2100
Shrub	*Lonicera ivolucrata*	13.1	2400
Shrub	*Cornus sericea*	12.2	2700
Shrub	*Rosa acicularis*	11.4	2800
Shrub	*Salix* spp.	9.1	1300
Forb	*Vicia sativa*	26.4	2400
Forb	*Urtica dioica*	25.8	2300
Forb	*Epilobium* spp.	22.2	2600
Forb	*Lathyrus* spp.	20.8	2300
Forb	*Geum rivale*	20.1	2400
Forb	*Dracocephalum parviflorum*	18.8	2000
Forb	*Viola* spp.	18.1	2200
Forb	*Martensia paniculata*	18.0	1800
Forb	*Prosartes* spp.	18.0	1600
Forb	Thalictrum venulosum	17.8	2400
Forb	*Galium* spp.	15.6	2100

Forb	*Equisetum* spp.	15.5	2000
Forb	*Lilium* spp.	14.5	2400
Forb	*Mitella diphylla*	13.2	2400
Forb	*Cornus canadensis*	9.8	2600
Forb	*Gaultheria* spp.	9.4	2600
Forb	*Linnaea borealis*	6.1	2100
Graminoid	Poaceae family	14.7	2300
Graminoid	*Carex* spp.	12.4	2100
