# Supplementary material for: The Role of Forage Quantity and Quality in the Migration and Diet of a Northern Ungulate During Their Neonatal Period
Source: Ecol Evol. 2026 Apr 8;16(4):e73454. doi: 10.1002/ece3.73454 (PMC13062649; doi:10.1002/ece3.73454)
Supplement: Supplementary file 8 — Appendix S8: Seasonal dietary contribution percents of forage groups for the Ronald Lake Wood Bison Herd, including all identified taxa with no exclusion threshold applied (i.e., taxa that accounted for at least 1% of the diet). [file ECE3-16-e73454-s002.pdf]

**Appendix 8.** Seasonal dietary contribution percents of forage groups for the Ronald Lake Wood Bison Herd, including all identified taxa with no exclusion threshold applied (i.e., taxa that accounted for at least 1% of the diet).

| Season | Browse (%) | Forb (%) | Graminoid (%) | Other (%) |
|---|---|---|---|---|
| Winter | 49.78 | 4.78 | 43.34 | 2.10 |
| Late Spring/ Early Summer | 84.88 | 12.92 | 0.09 | 2.11 |
| Mid-Late Summer | 60.23 | 36.47 | 0.50 | 2.80 |
